# Cooperative Effect of Multiple Domains in Copper Proteins Applied to H_2_S Sensing

**DOI:** 10.1021/acsomega.5c12638

**Published:** 2026-03-11

**Authors:** Alessio Trerotola, Viktoriia Vykhovanets, Lionel A. Ndamba, Daniela Guarnieri, Valeria Lagostina, Mario Chiesa, Stefano Milione, Maria Strianese

**Affiliations:** † Dipartimento di Chimica e Biologia “Adolfo Zambelli”, Università degli Studi di Salerno, Via Giovanni Paolo II, 132, 84084 Fisciano (SA), Italy; ‡ Leiden Institute of Physics (LION), Leiden University, P.O. Box 9504, 2300 RA Leiden, Netherlands; § Dipartimento di Chimica, 9314 Università di Torino, via Pietro Giuria 7, I-10125 Torino (TO), Italy

## Abstract

The revolutionary discovery by Abe & Kimura that H_2_S exerts a beneficial role in human body has renewed interest in this small molecule, long known for its toxicity. Understanding the (bio)­reactivity of H_2_S with biological and bioinorganic targets is therefore of increasing importance, yet studies on its interaction with nonheme metalloproteins remain limited. Here, we investigate the reactivity of HS^–^ with two natural multicopper proteins, SLAC and NiR. We demonstrate that SLAC, a two-domain blue-copper oxidase, can function as a multiwavelength, multireadout fluorescent sensor for H_2_S in complex environments. Comparative studies on NiR support the proposed mechanism of H_2_S recognition via selective reduction of copper centers. Finally, we benchmark the performance of these multicopper proteins against Cu-azurin, previously reported as a H_2_S recognition element, highlighting the advantages of multicopper architectures in terms of sensitivity, selectivity, and reversibility. Our findings establish multicopper proteins as versatile platforms for H_2_S sensing with potential applications in biomedical and environmental monitoring.

## Graphical abstract

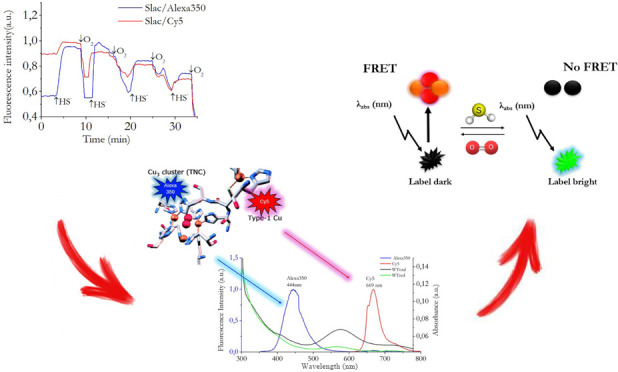

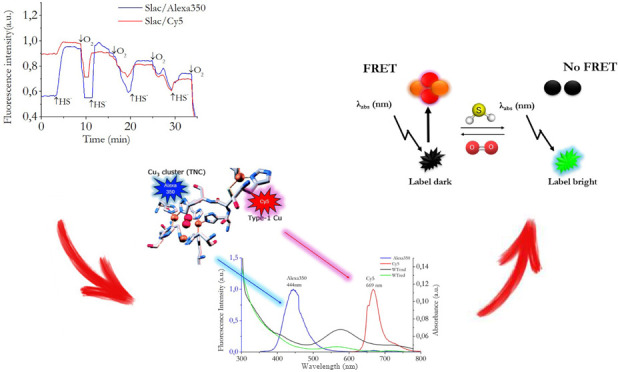

## Introduction

The traditional view of H_2_S is that of toxic gas capable of causing respiratory distress, eye irritation and, at high concentrations, unconsciousness or death.^1,2^ It poses particular risks in workplaces such as refineries, sewage, and mining plants. H_2_S also corrodes metals and infrastructure leading to costly damage in pipelines and other equipment.

Conversely, since the pioneering work of Abe and Kimura, it is well established that H_2_S, at low concentrations, functions as a biological signaling molecule in humans and other organisms.^3–6^ H_2_S donors and modulators are being explored as therapeutic agents for cardiovascular diseases, neuroprotection, and inflammation.^7,8^ In nature, H_2_S participates in the sulfur cycle and nutrient recycling. Physiological responses are typically triggered by H_2_S concentrations in the range 10 μM to 1 mM,^9^ although the intracellular H_2_S levels generated in response to physiological stimuli are still controversial. Furthermore, mechanistic understanding is still limited due to the fact that H_2_S is a weak acid, which in aqueous solution equilibrates with HS^–^ and S^2–^ thus complicating studies of protonation-state-dependent reactivity.^3–6^ At 20 °C, it has a p*K*_a1_ of 6.88 and a second p*K*_a2_ of 14.15, which is why, at the physiological pH of 7.4, about one-third exists as undissociated hydrogen sulfide and the remainder is largely the monohydrogen sulfide anion (the concentration of the completely deprotonated S^2–^ is extremely low).^10,11^

Within this context, our research focuses on elucidating H_2_S reactivity and developing efficient H_2_S/HS^–^ sensors. Our studies on H_2_S/HS^–^ coordination to transition metals have centered on tailored low-molecular-weight metal complexes.^12–18^ A common strategy for H_2_S/HS^–^ detection relies on activity-based fluorescent probes^19–22^ that generate a fluorescence signal upon H_2_S-induced chemical modification.^4,20,23–28^

Along with *ad-hoc* synthesized low molecular weight metal complexes,^21^ we and others have exploited the recognition properties of metalloproteins toward H_2_S.^29–34^

Metalloproteins offer defined redox and coordination environments that enable high specificity and sensitivity, alongside optical responses useful for signal transduction. Their biocompatibility further supports applications in biosensing and diagnostics.

Recently, we turned to copper proteins and found that the cupredoxin azurin^30^ can detect H_2_S/HS^–^ via a Förster resonance energy transfer (FRET) mechanism.^35–39^ Azurin is a member of the ubiquitous group of cupredoxins involved in the shuttling of electrons between proteins. The redox center is a type-I Cu­(II) center, responsible for the blue color.^40,41^

By several spectroscopic evidence, we found that the reaction of azurin with an excess of NaSH results in the reduction of the Cu­(II) center to Cu­(I). Upon HS^–^ addition, we observed loss of the blue color (typical of the type I copper center in the oxidized form), which is a further confirmation of the reduction of the copper center and renders azurin both a fluorescent and a colorimetric sensor for H_2_S.^30^ However, azurin lacks selectivity: glutathione (GSH) and l-cysteine (l-Cys) elicit similar responses, limiting its practical applicability.

To develop more selective sensing systems, we shifted our attention to a protein with a more complex copper active site: the small Laccase (SLAC). This enzyme contains multiple copper centers with distinct geometric and redox properties, potentially offering improved selectivity for sulfur-containing analytes such as H_2_S.

SLAC, a two-domain member of the laccase (four-copper oxidase) family, lacks the substrate-binding domain typical of three-domain laccases. We used SLAC from *Streptomyces coelicolor*, which has been extensively characterized by UV–vis, EPR, and NMR.^42^ Each monomer contains four copper ions organized into a type-I (T1) site and a trinuclear cluster (TNC; type-2 + two type-3 copper ions; Scheme 1). The T1 center (His_2_–Cys–Met ligation) oxidizes substrates, while the TNC accepts the transferred electrons to reduce O_2_ to H_2_O.

**Scheme 1:**
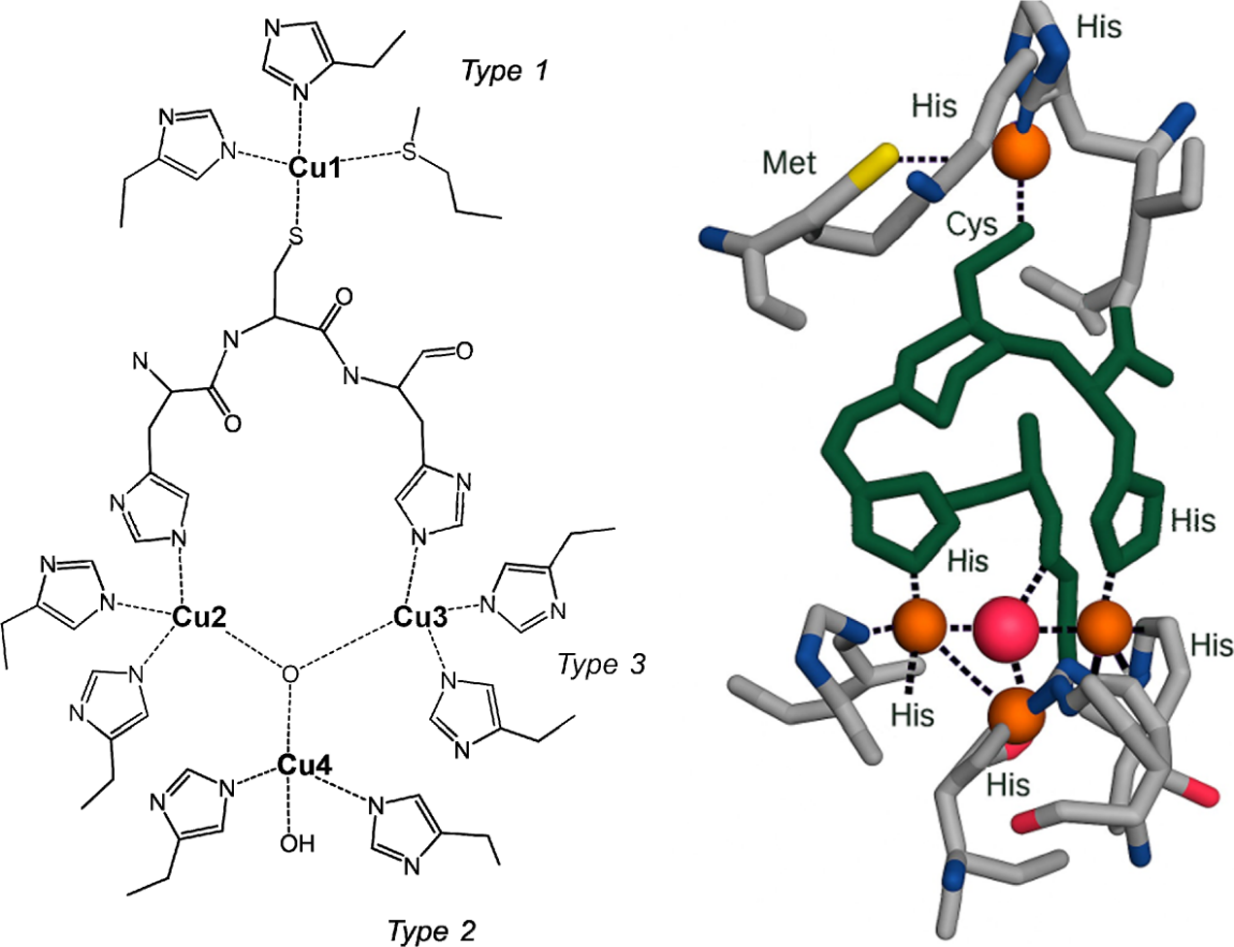
Schematic Representation of the Copper Sites in SLAC

Laccases exhibit broad substrate scope, efficient redox activity, high stability, and compatibility with diverse signal-transduction methods, supporting their use in applications ranging from bioremediation to biosensing.^43–45^

These attributes motivated our selection of SLAC to evaluate whether the cooperative action of its copper centers affords improved H_2_S sensing ability relative to azurin.

To further probe the role of multicopper architectures, we also investigated nitrite reductase (NiR) from *Alcaligenes faecalis* as a H_2_S sensor using the same FRET strategy. NiRs are structurally similar to SLAC, consisting of trimeric assemblies of two-domain monomers containing T1 and T2 copper sites, with the T2 center functionally replacing SLAC’s TNC.^46^

Finally, we performed imaging experiments in living cells to explore the applicability of these metalloprotein-based systems for H_2_S detection in complex biological environments.

## Results and Discussions

In the first series of experiments, we studied the reactivity of HS^–^ with SLAC via UV–vis spectroscopy. Native oxidized SLAC displays a characteristic blue color arising from a cysteine-to-copper LMCT transition at the T1 site, with an additional low-energy d–d contribution in the near-IR. Its UV–Vis spectrum features two diagnostic bands: a 320 nm band associated with the oxidized TNC, specifically the binuclear T3 center^47–50^ and a more intense band at 600 nm, characteristic of the oxidized T1 copper site (see Figures 1 and S1). Both bands are absent in the fully reduced, colorless enzyme, as reported either by adding dithionite (DT)^47^ or hydroquinone or ascorbate.^50,51^

**Figure 1:**
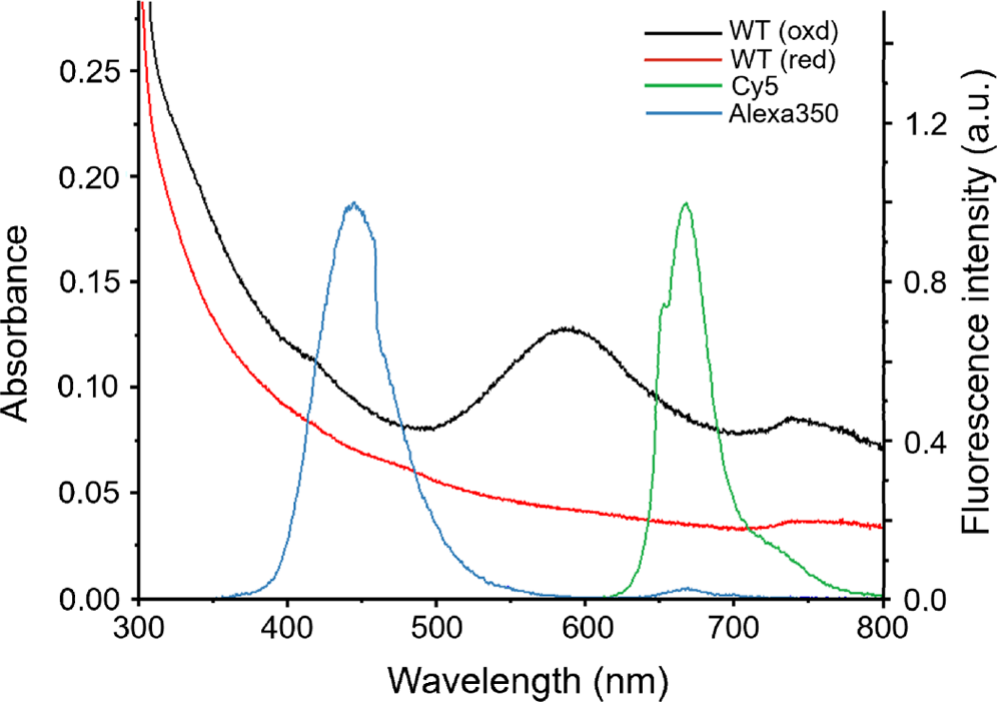
Absorption spectra of SLAC before (black trace) and after (red trace) HS^–^ addition and emission spectra of Alexa350 (λ_max_ = 444 nm) (blue trace) and of Cy5 (λ_max_ = 669 nm) (green trace). All spectra measured at room temperature. Protein concentration: 35 μM in 100 mM potassium phosphate buffer (pH = 6.8).

Upon addition of 50 μM of NaHS (HS^–^) both the 320 and 600 nm bands disappear (see Figure 1, red trace) accompanied by an immediate loss of the blue color observable by the naked eye.

One possibility was that HS^–^ displaces copper from SLAC, forming insoluble CuS and leaving a colorless apoprotein. However, bubbling oxygen into the HS^–^-treated sample restored both the original absorption spectrum and the blue color. The reversibility of these features, together with the absence of any black CuS/Cu_2_S precipitate, indicates that HS^–^ reduces the copper centers rather than removing them. These optical responses offered an opportunity to monitor the reactivity of each copper domain with HS^–^ and to assess whether SLAC can function as a multiwavelength or multireadout H_2_S sensor.

A further optical characteristic of SLAC is the fluorescence of the five tryptophans (Trps) at around 338 nm. In the oxidized state, this emission is quenched (∼70%) by FRET to the 320 nm TNC absorption band.^47^ We therefore monitored Trp fluorescence over time in the presence of HS^–^ and oxygen. As shown in Figure 2, HS^–^ reduces the TNC, leading to an increase in Trp fluorescence; reoxidation by oxygen restores the 320 nm band and reinstates FRET, returning fluorescence to its initial level. Most likely, the reason of the different rates during the subsequent cycles of red/ox is that going through one or more of these cycles activates the enzyme.^47^

**Figure 2:**
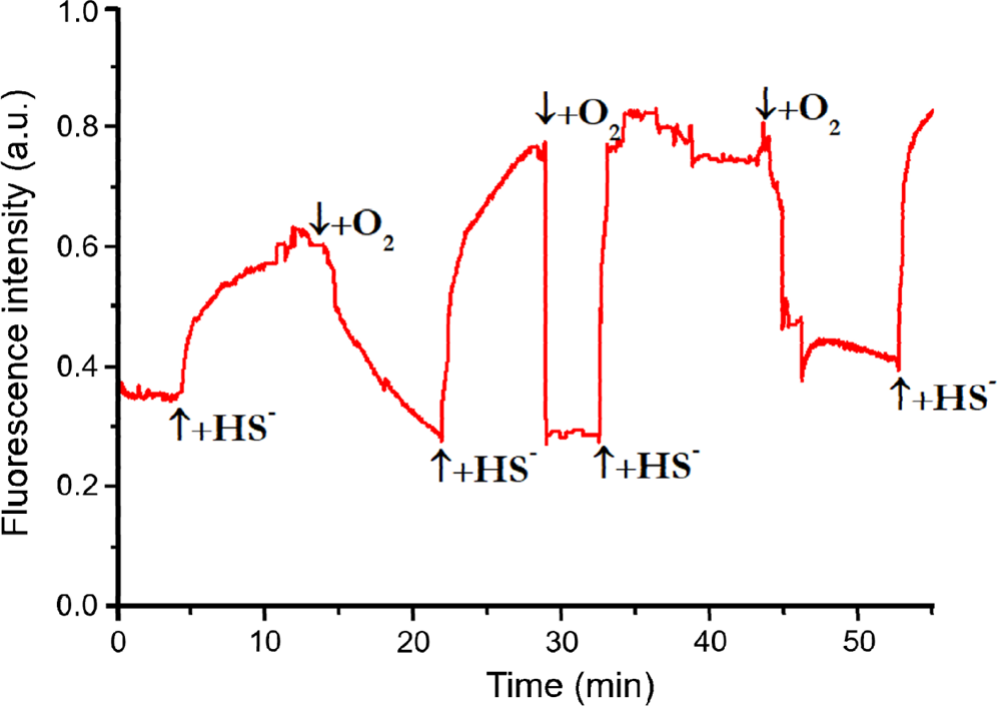
Fluorescence time trace for a solution containing unlabeled SLAC upon subsequent addition of NaSH (50 μM) and of an oxygen flow. Protein concentration: 20 μM in 100 mM potassium phosphate buffer (pH = 6.8); λ_ex_ = 280, λ_em_ = 338 nm.

However, near-UV fluorescence is difficult to measure in scattering media, and produces unstable signals (Figure 2). Moreover, this readout would be unsuitable in samples containing other near-UV-emitting species, whose background fluorescence could overwhelm the signal.^38^

To achieve a more robust and useful response, we covalently attached fluorescent labels (e.g., Alexa350 or Cy5) to the protein, with emission spectra partially overlapping either the 320 nm (TNC) or 600 nm (T1) absorption bands (Figure 1). This setup allowed us to probe whether the label fluorescence is modulated by HS^–^ via a FRET mechanism. In the absence of HS^–^ (oxidized SLAC), FRET from the label to the copper centers quenches fluorescence. When HS^–^ reduces the protein, the absorption bands disappear, FRET is switched off, and label emission is restored. Because the fluorescence response is governed by FRET, it selectively reports on the copper site whose absorption band overlaps with the emission of the attached fluorophore. Consequently, the absence of a fluorescence response does not necessarily indicate lack of reduction, but may reflect selective reduction of a copper domain that is not involved in the relevant FRET pathway. Labeling followed established protocols.^35,37,39^ and successful functionalization was confirmed by UV–Vis spectroscopy (Figures S2–S4).

Fluorescence of Alexa350-labeled SLAC (140 nM, excited at 350 nm) was monitored upon addition of NaSH (Figure 3). Each HS^–^ addition produced a clear fluorescence increase, which reverted upon oxygen exposure, demonstrating that TNC reduction by HS^–^ is reversible. Repeated cycles were possible, though maximum and minimum fluorescence gradually decreased, likely due to partial SLAC denaturation during O_2_ bubbling. Notably, the fluorescence of the reduced construct remained stable for hours when left undisturbed.

**Figure 3:**
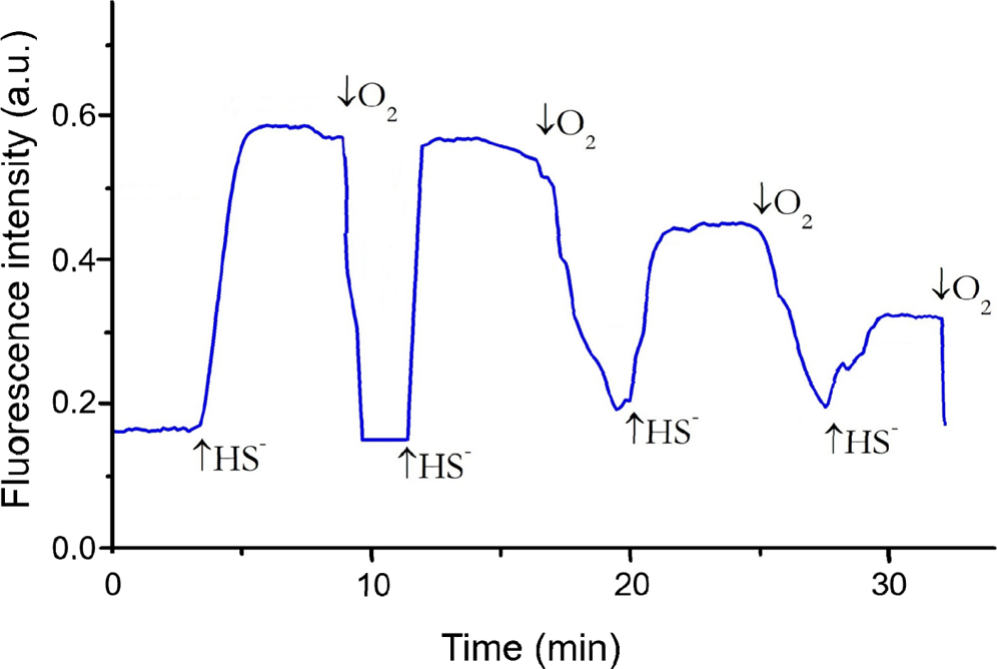
Fluorescence time trace for a solution containing Alexa350-labeled SLAC upon subsequent addition of NaSH (50 μM) and of an oxygen flow. Protein concentration: 140 nM in 100 mM potassium phosphate buffer (pH = 6.8); λ_ex_ = 350, λ_em_ = 444 nm. The first arrow indicates the time of injection of NaSH into the cuvette sample solution, the second arrow marks the time point at which O_2_ was introduced into the solution.

Monitoring the fluorescence of Cy5-labeled SLAC in the presence of excess NaSH revealed similar modulation, consistent with FRET between the 600 nm absorption band of SLAC and Cy5, and indicating that HS^–^-induced reduction of T1 is also reversible. Figure S5 shows a fluorescence time trace of Cy5-labeled SLAC (excitation at 651 nm).

Fluorescence intensities of SLAC_Alexa350 and SLAC_Cy5 increased with rising HS^–^ concentrations, showing a clear dose–response relationship (Figures S6 and S7). This enhancement reflects FRET “switch-off” from Alexa350 to the TNC and from Cy5 to T1, as previously observed. A detection limit in the micromolar range was determined (Figure S8).

An alternative fluorescence approach leverages FRET from Trp residues to a dye. When SLAC is labeled with a fluorophore absorbing around 330 nm, excitation of Trps at 280 nm transfers energy to the dye, generating visible emission: a strategy similar to the “Trp-to-dye” method reported by the Canters group for oxygen sensing.^38^ Figure 4 shows the fluorescence spectrum of Alexa350-labeled SLAC excited at 280 nm. Two bands are observed: Trp emission at 334 nm and Alexa350 emission at 442 nm via FRET.

**Figure 4:**
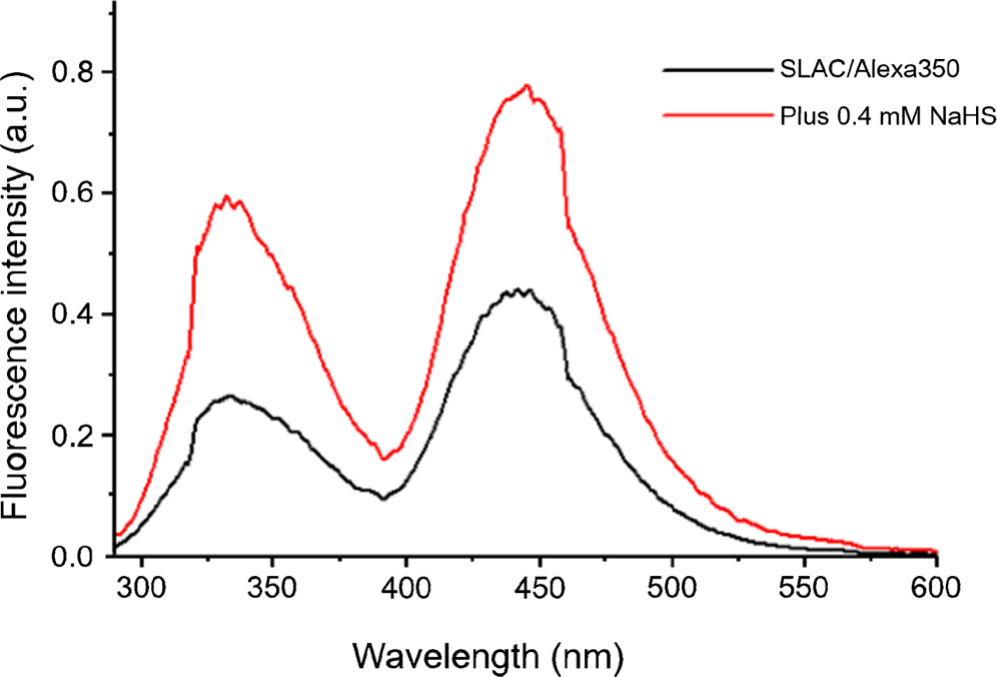
Room temperature fluorescence intensity traces (λ_exc_ 280 nm) of Alexa350 labeled SLAC before HS^–^ (black trace) and upon interaction with HS^–^ (red trace). Protein concentration: 120 nM in 100 mM potassium phosphate buffer (pH = 6.8).

The FRET channel is enabled by the spectral overlap between the Trps emission and Alexa350 absorbance (Figure S9). Both emissions increase upon HS^–^ addition, consistent with the other FRET-based readouts.

Collectively, these results demonstrate that the SLAC-based HS^–^ sensor can operate at three excitation/emission wavelength pairs (λ_ex = 280, 350, 651 nm; λ_em = 333, 442, 670 nm), making it a versatile platform suitable for diverse applications in complex biological environments.

### Selectivity of SLAC toward HS^–^

To assess the selectivity of the SLAC-based sensor, Alexa350- or Cy5-labeled SLAC was titrated with increasing concentrations of glutathione (GSH) or l-cysteine (l-Cys). In both cases, no significant fluorescence changes were observed, in stark contrast to the clear responses seen with HS^–^ (see Figure 5 vs Figures S6 or S7). This demonstrates that the SLAC-based sensor selectively recognizes HS^–^ over other biologically relevant thiols.

**Figure 5:**
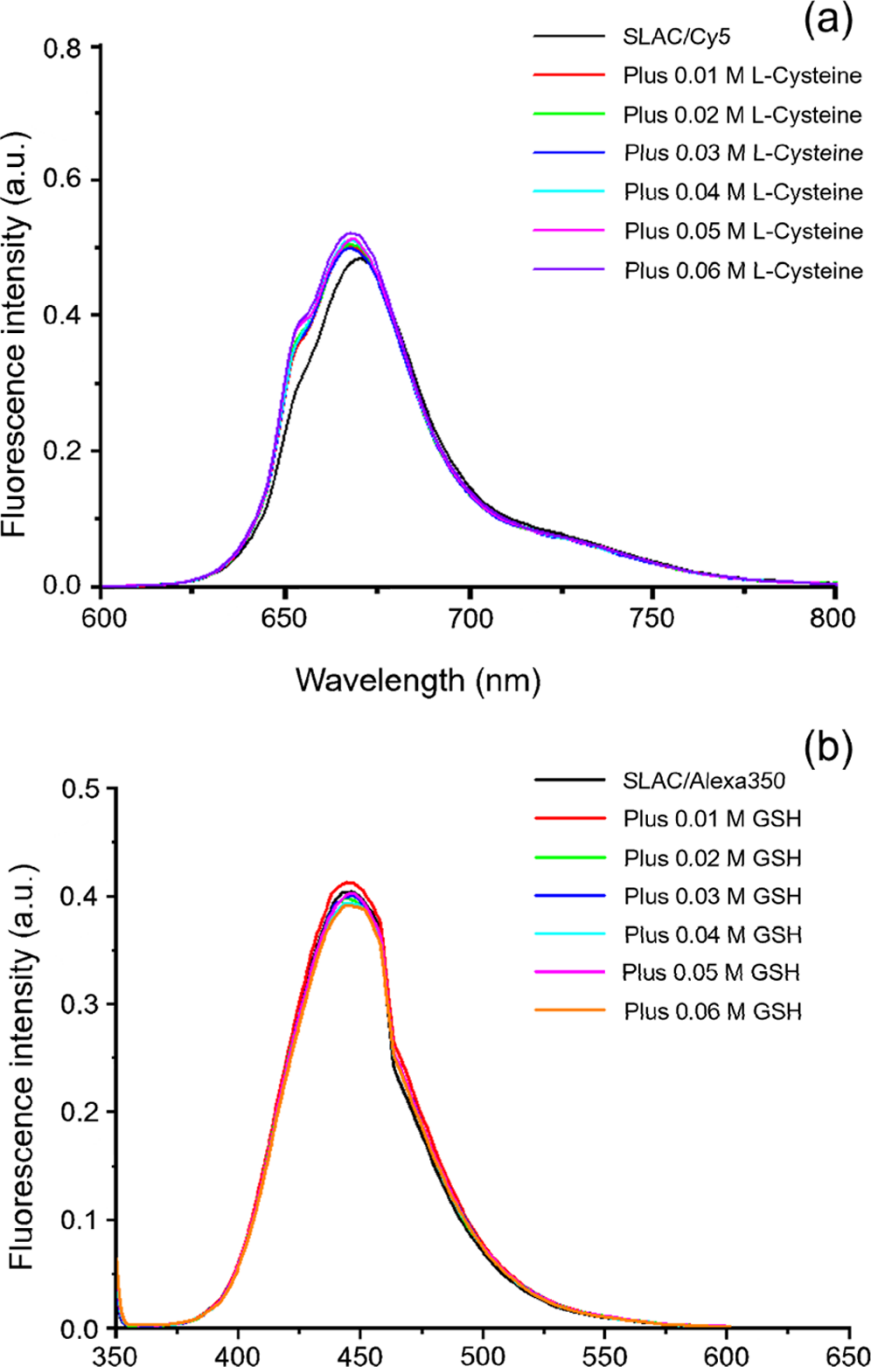
Room temperature fluorescence intensity traces of Cy5 labeled SLAC (exc 651 nm) titrated with l-Cys (a) and of Alexa350 labeled SLAC (exc 343 nm) titrated with GSH (b). Protein concentration: 120 nM in 100 mM potassium phosphate buffer (pH = 6.8).

The observed selectivity likely arises from the redox properties of SLAC. Unlike our previously reported azurin-based system, SLAC is preferentially reduced by HS^–^ rather than GSH or l-Cys. This finding is in line with the approximate standard formal reduction potentials (GSH = −240 mV; l-Cys = −223 mV; HS^–^ = −270 mV) which are reported in the literature.^11,52,53^

Laccases are commonly characterized by the midpoint potential of their type-1 copper site, distinguishing high-potential (∼800 mV) and low-potential (∼400 mV) enzymes.^54^ SLAC from *S. coelicolor* exhibits a midpoint potential of 378 ± 5 mV, classifying it as a low potential enzyme.^47,48,55,56^ However, for a precise understanding of the redox behavior of the enzyme, the presence of the TNC domain in SLAC cannot be ignored as it likely contributes to the enhanced selectivity toward H_2_S compared to azurin. To independently confirm SLAC reduction by HS^–^ and further investigate its selective response, we employed EPR spectroscopy.

The X-band CW EPR spectrum of oxidized SLAC, in frozen solution, displays a complex pattern indicative of at least two distinct Cu­(II) sites (Cu_1_ and Cu_2_, Figure 6). The binuclear T3 copper pair is EPR silent^57,58^ and does not contribute to the CW-EPR spectra and is therefore not discussed here; information on the redox state of the T3 center is instead obtained from the UV–Vis spectra (vide supra). Two sets of four Cu hyperfine lines are observed centered at *g*_∥_ ≈ 2.26 (Cu_1_) and *g*_∥_ ≈ 2.24 (Cu_2_), with differing hyperfine couplings (*A*_∥_(Cu_1_) ≈ 580 MHz, *A*_∥_(Cu_2_) ≈ 280 MHz). These values are consistent with those previously reported for Cu^2+^ T_1_ and Cu^2+^ T_2_ centers (of TNC) in the resting oxidized form of SLAC (WT, Y108F).^59^

**Figure 6:**
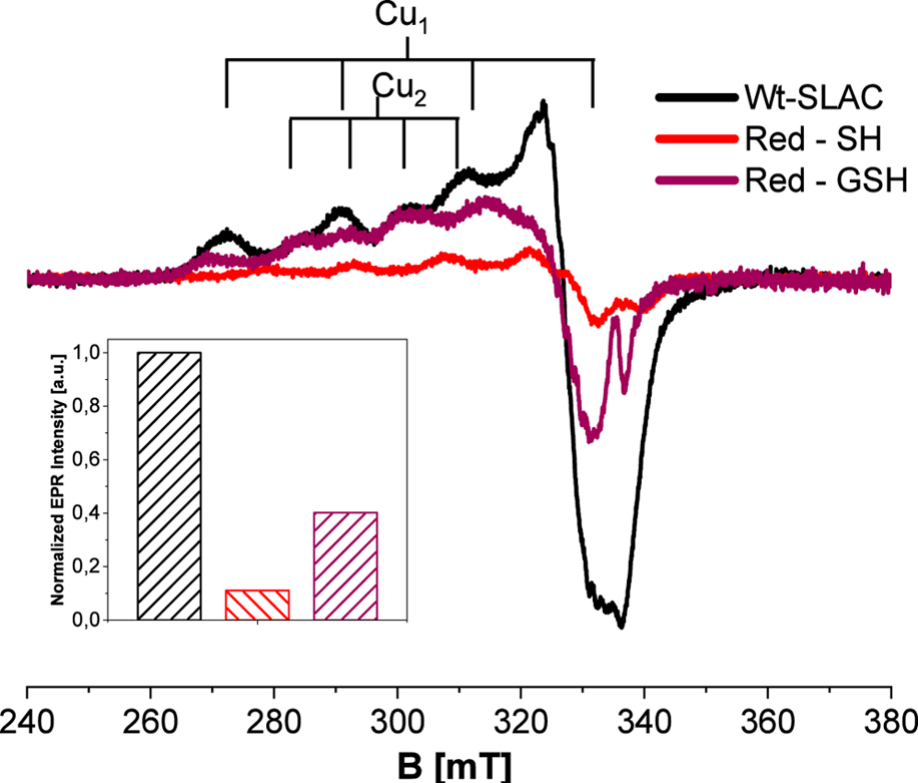
CW-X-band EPR spectra recorded at 77 K of the oxidized Wt-SLAC (black) and reduced with SH (red) and GSH (purple). The signal intensities reported in the inset were obtained by double integration of the CW-EPR spectra. Protein concentration: 0.13 mM with 30% glycerol.

Upon treatment with NaSH, EPR intensity decreases by ∼90% (inset, Figure 6), indicating substantial reduction of both Cu­(II) species. In contrast, GSH produces a smaller decrease. Double integration of the spectrum (purple trace in Figure 6) indicates a reduction of ∼60% of the original intensity. The residual signal displays *g*_∥_ and *A*_∥_ values characteristic of the Cu­(II) T2 site (see stick diagram in Figure 6), indicating that the remaining EPR signal mainly originates from CuT2. Since CW-EPR selectively probes paramagnetic Cu­(II) centers, mainly T1 and T2, the observed decrease in EPR intensity reflects partial reduction of these sites and does not provide information on the redox state of the T3 pair, which is EPR silent due to strong antiferromagnetic coupling.

In contrast, GSH produces a smaller decrease (∼60%), leaving a residual signal mainly associated with Cu_2_.

These data indicate that GSH is a milder, more selective reductant, preferentially targeting Cu1, whereas HS^–^ efficiently reduces both copper sites. Taken together, the EPR and fluorescence data are fully consistent when their intrinsic site selectivity is taken into account: agreement between the two techniques is observed when they probe the same copper domain, whereas apparent discrepancies arise when EPR and fluorescence are sensitive to different sites.

In line with these findings if titrating Cy5-labeled SLAC with increasing amounts of GSH (looking at the response of the Cu^2+^ T_1_ domain) a pronounced enhancement of the fluorescence intensity was observed (see Figure S10) differently than that observed with Alexa350-labeled SLAC (see Figure 5).

To further probe selective T2 reduction, we studied another multicopper protein, nitrite reductase (NiR) from *A. faecalis*. NiR catalyzes the reduction of nitrite (NO_2_^–^) to nitric oxide (NO), a key step in the nitrogen cycle. It contains a type-1 site, which accepts electrons from donors such as azurin, and a type-2 site, where nitrite is reduced (see Figure S11).^60^

The T2 site is coordinated by three histidines and a water or hydroxide ligand in a pseudotetrahedral geometry, optimized for substrate binding and reduction. Electrons flow from T1 to T2 to enable catalysis.

EPR analysis of NiR exposed to HS^–^ and GSH shows a pattern analogous to SLAC: HS^–^ induces near-complete reduction of both Cu­(II) sites, whereas GSH produces a more limited effect (Supporting Information Figure S12). The same behavior observed for SLAC and NiR highlights that multicopper centers can be exploited to achieve selective, reversible detection of H_2_S. This finding implies that proteins with multiple copper domains, like SLAC or NiR, can provide both sensitivity and selectivity in metalloprotein-based H_2_S sensors, enabling multisite, multireadout detection strategies in complex environments.

Furthermore, we explored whether fluorescently labeled NiR responds to H_2_S in a similar manner. Upon each HS^–^ addition, a rapid increase in label emission was observed. Subsequent addition of K_3_Fe­(CN)_6_ restored the oxidized state of the protein and quenching of the fluorescence. In other words, the label switches ON in the presence of H_2_S and OFF when the protein is reoxidized (Figure S13). This cycle could be repeated multiple times, demonstrating that HS^–^-mediated reduction of the T2 center in NiR is fully reversible, which is an essential feature for practical sensing applications.

Finally, we explored the nature of the interaction between HS^–^ and the TNC in SLAC. In this context, HS^–^ coordination to the Cu­(II) center can be viewed as a prerequisite for electron transfer from HS^–^ to copper, thereby enabling the HS^–^-induced reduction of Cu­(II) to Cu­(I). Previous studies have shown that anions such as fluoride and chloride can bind to the type-2 copper ion, thereby inhibiting the enzyme’s activity.^61^ Using DFT calculations, Kepp, suggested that such binding increases the reorganization energy of the second reduction step and lowers the reduction potential compared to the hydroxide-bound resting state.^62^ By analogy, HS^–^ might coordinate similarly. Using a DFT model of the oxidized TNC, we located an adduct in which HS^–^ binds the type-2 copper (Figure 7), indicating that HS^–^ binding to the type-2 copper is structurally plausible.

**Figure 7:**
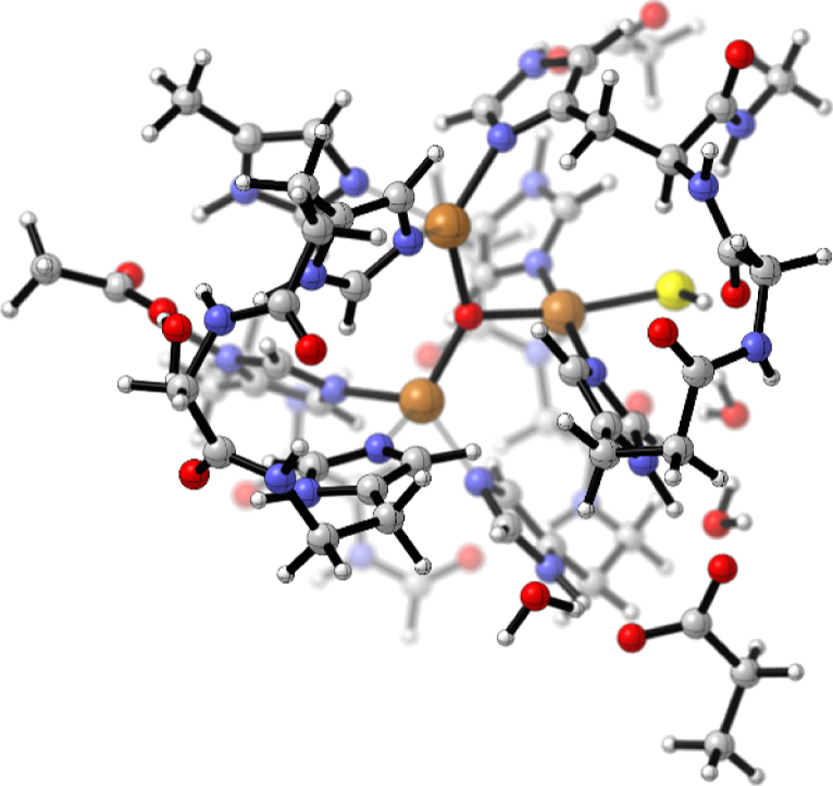
Geometry-optimized structures of the TNC model showing the coordination of the HS^–^ anion to the to the type 2 copper ion.

Computed ionization energies for partially oxidized states of this adduct were very similar to those of the native OH^–^-bound form. Thus, while HS^–^ can coordinate to the T2 site, this interaction does not significantly alter its reduction behavior.

### Biological Assays in Living Cells

Encouraged by the fluorescence response of SLAC to HS^–^ in vitro, we explored the potential of fluorescently labeled SLAC to visualize exogenous H_2_S in HepG2cells.

Prior to imaging, cytotoxicity of the probe was evaluated using an MTT assay (3-(4,5-dimethylthiazol-2-yl)-2,5-diphenyltetrazolium bromide, Sigma-Aldrich). HepG2 cells treated with SLAC_Cy5 showed only a slight, nonsignificant decrease in viability compared to untreated controls, indicating negligible toxicity under the experimental conditions (Figure S14).

For imaging experiments, cells were incubated with the probe for 60 min. As shown in Figure 8C, cells displayed enhanced fluorescence relative to untreated controls (panels A and B). To test the ability of SLAC_Cy5 to detect exogenous H_2_S, cells were incubated with the probe and then treated with 260 μM NaSH, a concentration comparable to physiological levels. This treatment produced a further, pronounced increase in fluorescence (Figure 8D), demonstrating that the probe can effectively sense HS^–^ within live cell environments and more complex biological fluids. Despite our tentative, we did not find conditions that would enable this system to serve as a reversible sensor of H_2_S in cellular environments.

**Figure 8:**
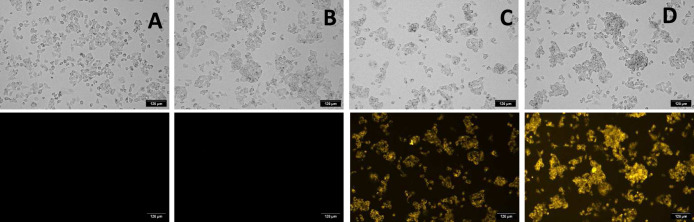
Fluorescence microscopy images of nontreated HepG2 cells (A), of HepG2 cells treated with PBS (B), of HepG2 cells incubated with SLAC_Cy5 (C), of HepG2 cells incubated with SLAC_Cy5 + 260 μM NaSH (exogenous HS^–^) (D). Protein concentration: 120 nM. Magnification 20×.

As control experiment we performed the same experiment with bovine serum albumin (BSA) labeled with Cy5 and in the presence of HS^–^: no fluorescence raise upon addition of HS^–^ in cells was observed (Figures S15C,D).

## Conclusions

In this work we studied the interaction of HS^–^ with SLAC from *streptomyces coelicolor* with different spectroscopies and provided evidence that fluorescently labeled SLAC can serve as a reversible FRET-based sensor for H_2_S detection. We choose Alexa350 whose emission inversely reflects the intensity of the 320 nm absorption band (the TNC cluster) whereas Cy5 that of the 600 nm absorption band (the T1-center).

To support the hypothesis that HS^–^ binds the type-2 copper in the TNC center, we also examined fluorescently labeled NiR, which contains a T2 copper site analogous to SLAC’s TNC. NiR displayed a similarly reversible fluorescence response, highlighting the potential of multicopper proteins as efficient H_2_S FRET sensors.

Consistent with previous findings for Cu-azurin, HS^–^ reduces the Cu­(II) centers to Cu­(I), accompanied by loss of the characteristic blue color of the type-I copper domain, which is restored upon oxygenation. In the oxidized state, FRET from the copper centers partially quenches the fluorophore emission, while in the reduced state, quenching is relieved, resulting in enhanced fluorescence. This reversible behavior is advantageous for practical sensing applications, enabling repeated use of the same sensor.

The SLAC- and NiR-based sensors exhibit marked selectivity for HS^–^, and their multiwavelength fluorescence capability adds versatility for diverse measurement conditions. Importantly, we demonstrate here for the first time that an enzyme-based, multicopper FRET sensor can detect H_2_S/HS^–^ in living cells, representing a significant improvement over previously reported systems.

Overall, our results suggest that multicopper proteins can be developed as robust, selective, and reusable FRET-based sensors for H_2_S/HS^–^, with potential applications in both biomedical and environmental monitoring. Such protein-based approaches align with the growing interest in sustainable, biocompatible sensing technologies for H_2_S a molecule that, despite its toxicity, plays critical roles in human physiology and ecological cycles.

## Experimental Section

### Materials

All chemicals used for the synthetic work were obtained from Sigma-Aldrich. They were used without further purification. Atto620 NHS-ester was purchased from ATTO-TEC Biolabeling and Ultraanalytics (Siegen, Germany). Cy5 NHS-ester was purchased from Amersham Biosciences (Freiburg, Germany). Alexa 350 NHS-ester was purchased from molecular probes (Leiden, The Netherlands). Stock solutions of the dyes (50 mM) were prepared by dissolving the powders in water-free DMSO. Wild-type SLAC was expressed and purified as previously reported.^42^ NiR from *A. faecalis* was expressed and purified as described previously.^63^

### Protein Labeling

SLAC and NiR were incubated with amino-reactive labels (NHS esters) for amino labeling, using procedures reported in the literature^39,47,64^ (e.g., the most useful reaction for labeling at amino groups is acylation). More specifically, proteins were labeled with the fluorophores in a roughly 10 times molar excess over the protein concentration in potassium phosphate buffer 100 mM, pH 6.8 and incubated for about 2 h at room temperature in the dark. The unbound label was removed by two consecutive size-exclusion chromatography steps. Labeling ratios were in the range of 0.2–0.9 (dye molecule/protein), as can be easily determined from the absorption spectra of the labeled proteins (Figures S2–S4).

The dye over protein (DOL) ratio has been quantified as suggested by the manufacturers taking ε_645_ = 250 mM^–1^ cm^–1^ for Cy5, ε_346_ = 19 mM^–1^ cm^–1^ for Alexa350; ε_620_ = 120 mM^–1^ cm^–1^ for Atto620.

The concentration of proteins was determined optically at 280 nm using ε_280_ = 1000 M^–1^ cm^–1^ (SLAC) and ε_280_ = 46000 M^–1^ cm^–1^ (NiR).^63^

### Absorbance and Fluorescence Measurements

Absorption spectra were recorded on a Cary-50 spectrophotometer using a 1 cm quartz cuvette (Hellma Benelux BV, Rijswijk, Netherlands) and a slit-width equivalent to a bandwidth of 5 nm. Fluorescence spectra were measured on a Cary Eclipse spectrophotometer in a 10 × 10 mm^2^ airtight quartz fluorescence cuvette (Hellma Benelux BV, Rijswijk, Netherlands) with an emission band-pass of 10 nm and an excitation band-pass of 5 nm. Both absorption and fluorescence measurements were performed in potassium phosphate buffer 100 mM pH 6.8 at room temperature under aerobic conditions. Fluorescence emission spectra of the labeled proteins were registered by exciting the samples at the absorption maximum of the label (as specified in the figure caption). NaSH solutions in Milli-Q water were used as HS^–^ donors.

### EPR Spectroscopy

X-band (microwave frequency 9.46 GHz) CW EPR spectra were performed at 77 K on a Bruker EMX spectrometer equipped with a cylindrical cavity. A modulation frequency of 100 kHz, a modulation amplitude of 0.4 mT, and a microwave power of 1 mW were used. One ml of Wt-SLAC solution (*C* ≈ 0.13 mM) was prepared with 30% glycerol, ≈300 μL of solution were transferred into a 4 mm quartz EPR tube. The same procedure was used for the preparation of the NiR sample. From the protein solution two aliquots were taken, and NaSH or GSH were added in excess to them to reduce the proteins. Quantitative analysis of the EPR signal intensity was carried out by double integration of the first-derivative spectra using routines available in the Xepr software package (Bruker Biospin). Spectra were acquired at microwave power levels (1 mW) well below those at which significant saturation occurs, ensuring a linear relationship between signal intensity and spin concentration. Prior to integration, the spectra were baseline-corrected by polynomial fitting to ensure a constant baseline on both sides of the signal. The magnetic-field scan range was chosen to exceed the field region where the signal amplitude approached the noise level, ensuring that the full absorption profile was included in the integration, thereby minimizing truncation errors.

### Control Experiments

No significant changes in the fluorescence intensity were observed when monitoring labeled SLAC or labeled NiR in the absence of HS^–^. Neither did HS^–^ addition show any effect, when free dyes or Cy5_labelled BSA were used as sensing materials. Furthermore, the proposed sensors did not exhibit a change in fluorescence when bubbling argon through the solutions. In order to check any eventual interaction of the phosphate with the copper centers, as preliminary indication, we repeated the same titrations we had performed for the experiments displayed in Figures S6 and S7 but adding aliquots of a phosphate buffer much more concentrated than before (in the end the phosphate concentration was 200 mM). Again, no significant changes in the fluorescence intensity could be observed.

### Computational Details

Molecular structures and electronic energies were calculated using the Gaussian 0938 packages at the BP86/def2SVP level of theory. The model for the TNC center of the oxidized (RO) state of the SLAC was taken from ref 62. We modeled the fully oxidized states and in the one- or two-electron reduced states featuring both OH^–^ and SH^–^ ligand. Cartesian coordinates of all DFT-optimized structures are available upon request.

### MTT Assay

Cell viability was analyzed by 3-(4,5-dimethylthiazol-2-yl)-2,5-diphenyltetrazolium bromide (MTT; Sigma-Aldrich) assay. Then, 1.5 × 10^4^ cells were seeded in each well of a 96-multiwell plate. Twenty-four hours after cell seeding, HepG2 cells were incubated with SLAC_Cy5 obtained by eluting the labeled protein during size-exclusion chromatography in the cell culture medium. After 2 h of incubation with SLAC_Cy5 solutions, the MTT reagent was added to the cell media of each sample (final concentration 0.125 mg/mL) and incubated for 1 h at 37 °C. The resulting formazan crystals were dissolved in DMSO. Absorbance was measured at 570 and 690 nm wavelengths by a multiplate reader, and raw data were normalized to nontreated cells (considered 100%) to calculate cell viability percentage. Data were reported as mean ± standard deviation (*n* = 8).

### Cell Culture

HepG2 cells (Human hepatocellular liver carcinoma cell line) were grown in minimum essential medium (MEM) supplemented with 10% fetal bovine serum (FBS), 2 mM glutamine, 1 mM nonessential amino acids and 1% antibiotics (penicillin/streptomycin, 100 U/mL). Cells were maintained in a humidified incubator at 37 °C, in 5% CO_2_/95% air. 1.5 × 10^5^ cells/well were seeded on 12-well multiwell plates 1 day before imaging.

### Fluorescence Imaging

To verify the loading of the probe and capability in HS^–^ detection, HepG2 cells were incubated with labeled SLAC in PBS diluted in cell culture medium for 1 h at 37 °C. After incubation, cells were rinsed to remove excess of protein. Probe-loaded cells were observed by an automated inverted fluorescence microscope (Olympus IX83) at 545–557 nm excitation wavelength using a 10× objective and 20× objective. Only probe-loaded cells were further treated with exogenous NaSH (260 μM in MEM) for 30 min and then observed with the microscope to test the capability of SLAC to monitor the intracellular increase of HS^–^.

## Supplementary Material



## Notes

Absorption and fluorescence spectra of labeled SLAC (opj), fitting of fluorescence data (opj), schematic representation of NiR cofactor, EPR spectra of NiR, fluorescence time-trace spectrum of NiR (opj) cytotoxicity evaluation of SLAC_Cy5, fluorescence microscopy images of HepG2 cells before and after incubation with BSA_Cy5 (jpg) (PDF)
