# Supplementary material for: Cooperative Effect of Multiple Domains in Copper Proteins Applied to H2S Sensing
Source: ACS Omega. 2026 Mar 11;11(11):17977–85. doi: 10.1021/acsomega.5c12638 (PMC13019397; doi:10.1021/acsomega.5c12638)
Supplement: Supplementary file 1 [file ao5c12638_si_001.pdf]

The cooperative effect of multiple domains in copper proteins applied to H_2S sensing.

Alessio Trerotola[a], Viktoriia Vykhovanets[a], Lionel A. Ndamba[b], Daniela Guarnieri[a], Valeria Lagostina[c], Mario Chiesa[c], Stefano Milione[a], Maria Strianese.[a]*

[a]Dipartimento di Chimica e Biologia "Adolfo Zambelli", Università degli Studi di Salerno, Via Giovanni Paolo II, 132, 84084 Fisciano (SA) Italy; [b]Leiden Institute of Physics (LION), Leiden University, P.O. Box 9504, 2300 RA, Leiden, Netherlands; [c]Dipartimento di Chimica, Universita` di Torino, via Pietro Giuria 7, I-10125 Torino (TO), Italy.

*Corresponding author. E-mail: mstriane@unisa.it

Supporting information

Contents:

Figure S1	UV-vis spectrum of SLAC	S3
Figure S2	UV-vis spectrum of SLAC_Alexa350	S4
Figure S3	UV-vis spectrum of SLAC_Cy5	S5
Figure S4	UV-vis spectrum of SLAC Atto620	S6
Figure S5	Fluorescence time trace of Cy5-labelled SLAC	S7
Figure S6	Emission spectra of SLAC_Alexa350	S8
Figure S7	Emission spectra of SLAC_Cy5	S9
Figure S8	Linear best fit of calibration line for the calculation of LOD for HS^-	S10
Figure S9	Overlay of the UV-vis spectrum of Atto620 and of Trps emission	S11
Figure S10	Emission spectra of SLAC_Cy5	S12
Figure S11	Schematic representation of the copper sites in NiR	S13
Figure S12	CW-X-band EPR spectra of NiR	S14
Figure S13	Fluorescence time trace for Cy5-labelled NiR	S15
Figure S14	Cytotoxicity evaluation of SLAC_Cy5 in HepG2 cell line by MTT assay	S16
Figure S15	Fluorescence microscopy images of HepG2 cells incubated with BSA_Cy5	S17

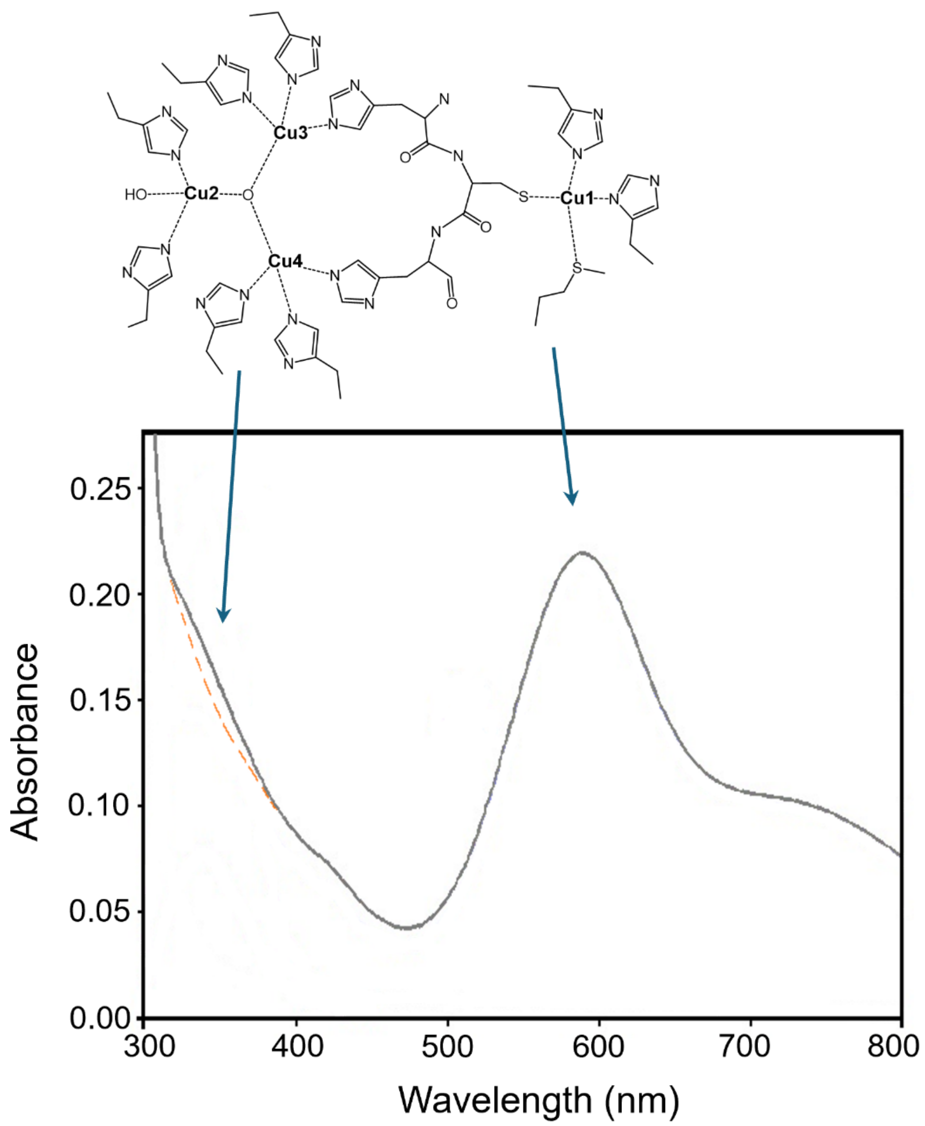

Figure S1. Electronic absorption spectrum of SLAC.

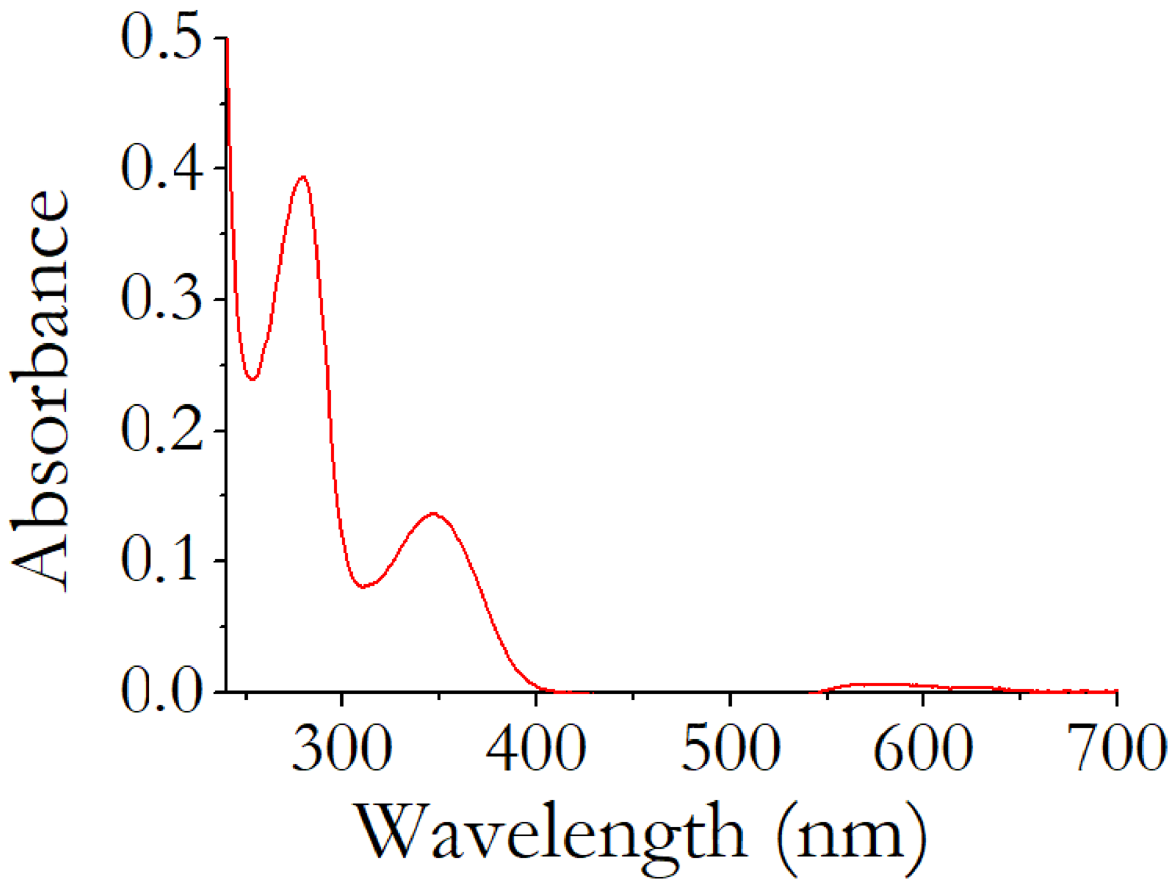

Figure S2. Electronic absorption spectrum of Alexa350 labelled SLAC. Protein concentration: 320 μM.

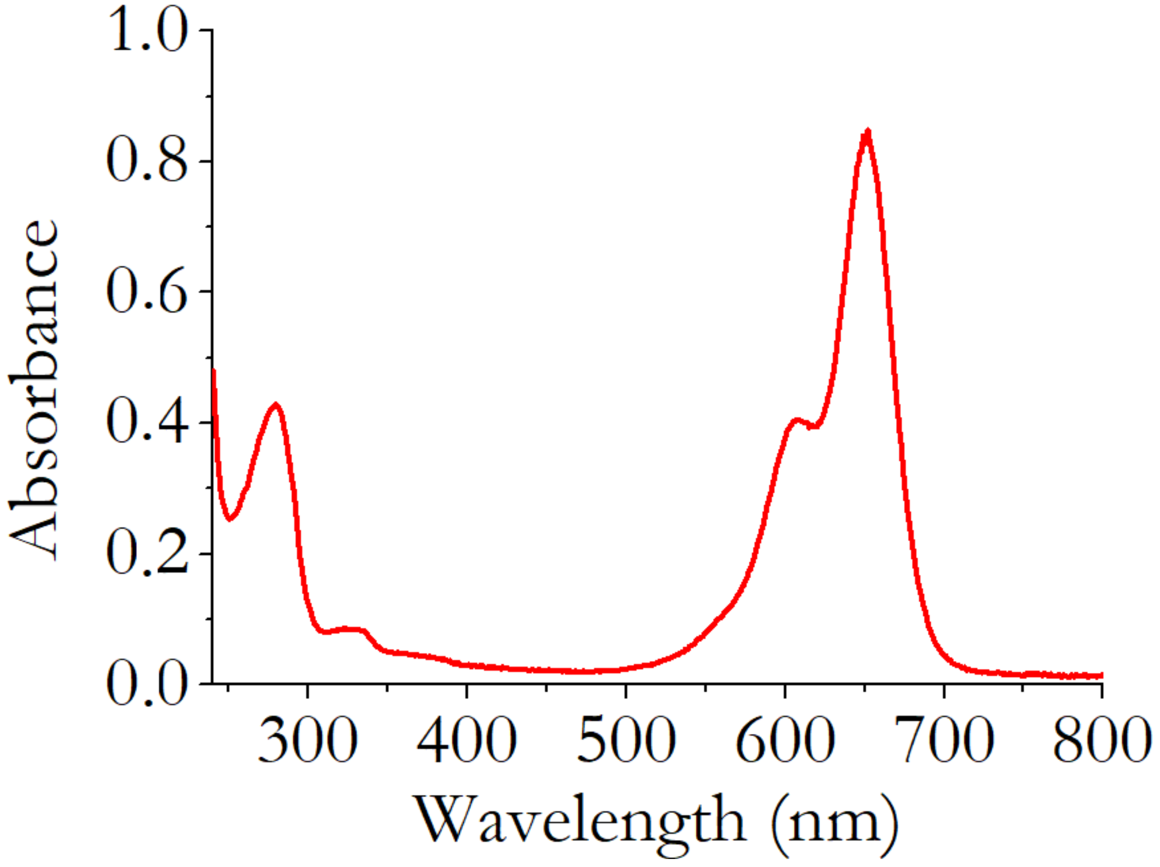

Figure S3. Electronic absorption spectrum of Cy5 labelled SLAC. Protein concentration: 340 μM.

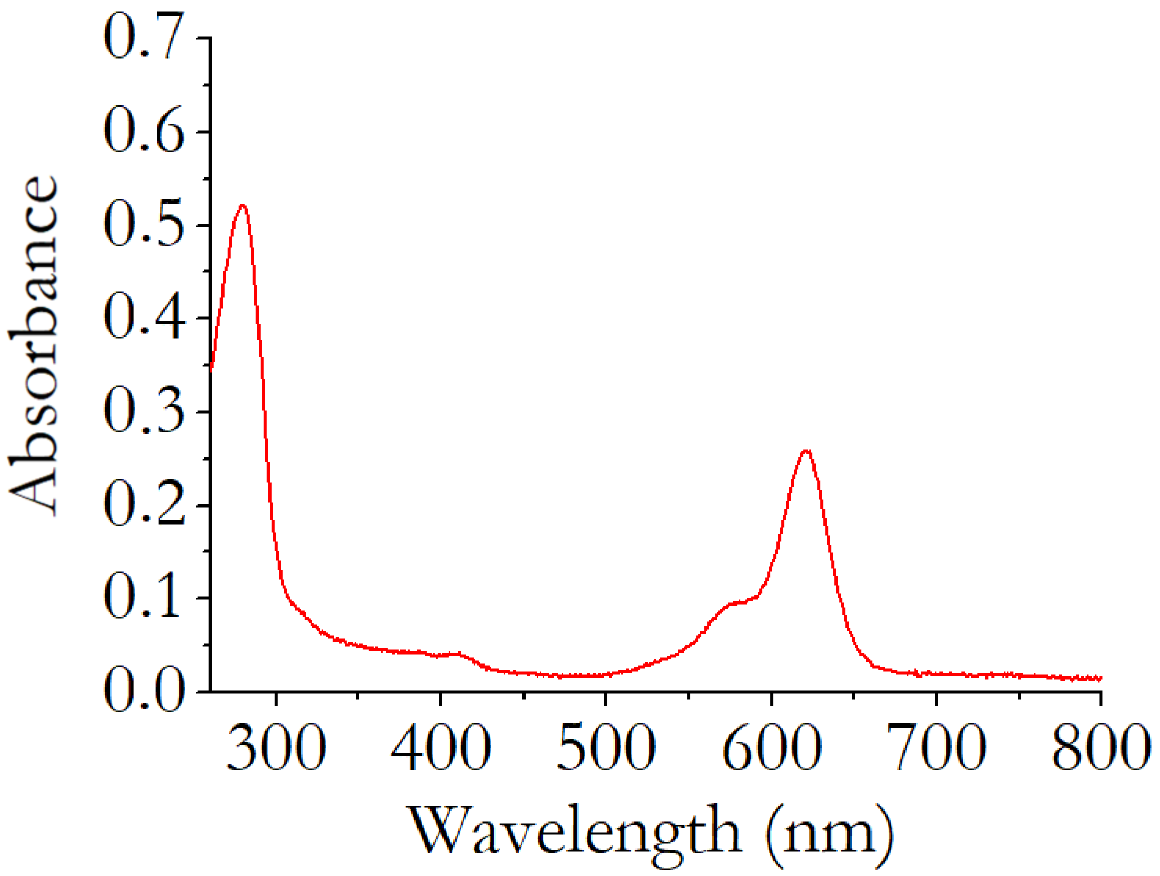

Figure S4. Electronic absorption spectrum of Atto620 labelled SLAC. Protein concentration: 320 µM.

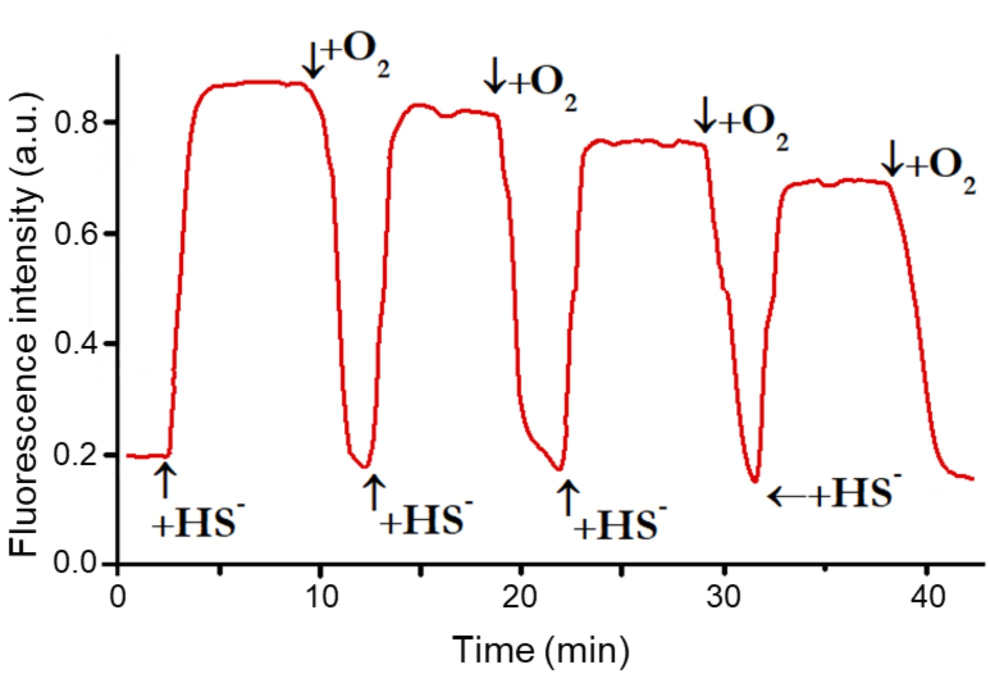

Figure S5. Fluorescence time trace for a solution containing Cy5-labelled SLAC upon subsequent addition of NaSH (50 µM) and of an oxygen flow. Protein concentration: 140 nM in 100 mM potassium phosphate buffer (pH = 6.8); λ_{ex} = 651, λ_{em} = 670 nm. The first arrow indicates the time of injection of NaSH into the cuvette sample solution, the second arrow marks the time point at which O_2 was introduced into the solution.

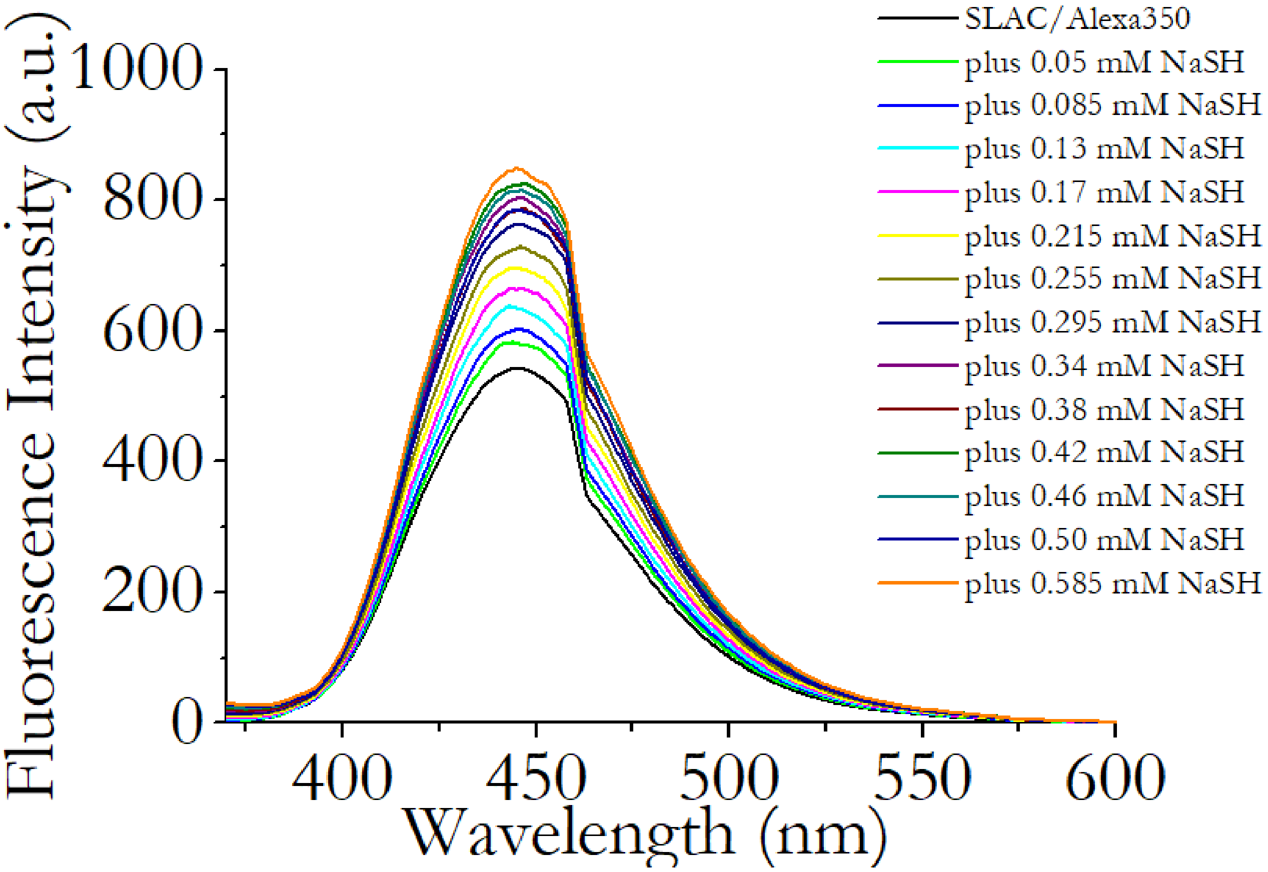

Figure S6. Emission spectra of SLAC_Alexa350 before and after the addition of increasing amounts of NaSH. 100 mM potassium phosphate buffer (pH = 6.8); λ_{ex} = 350 nm. Protein concentration: 140 nM.

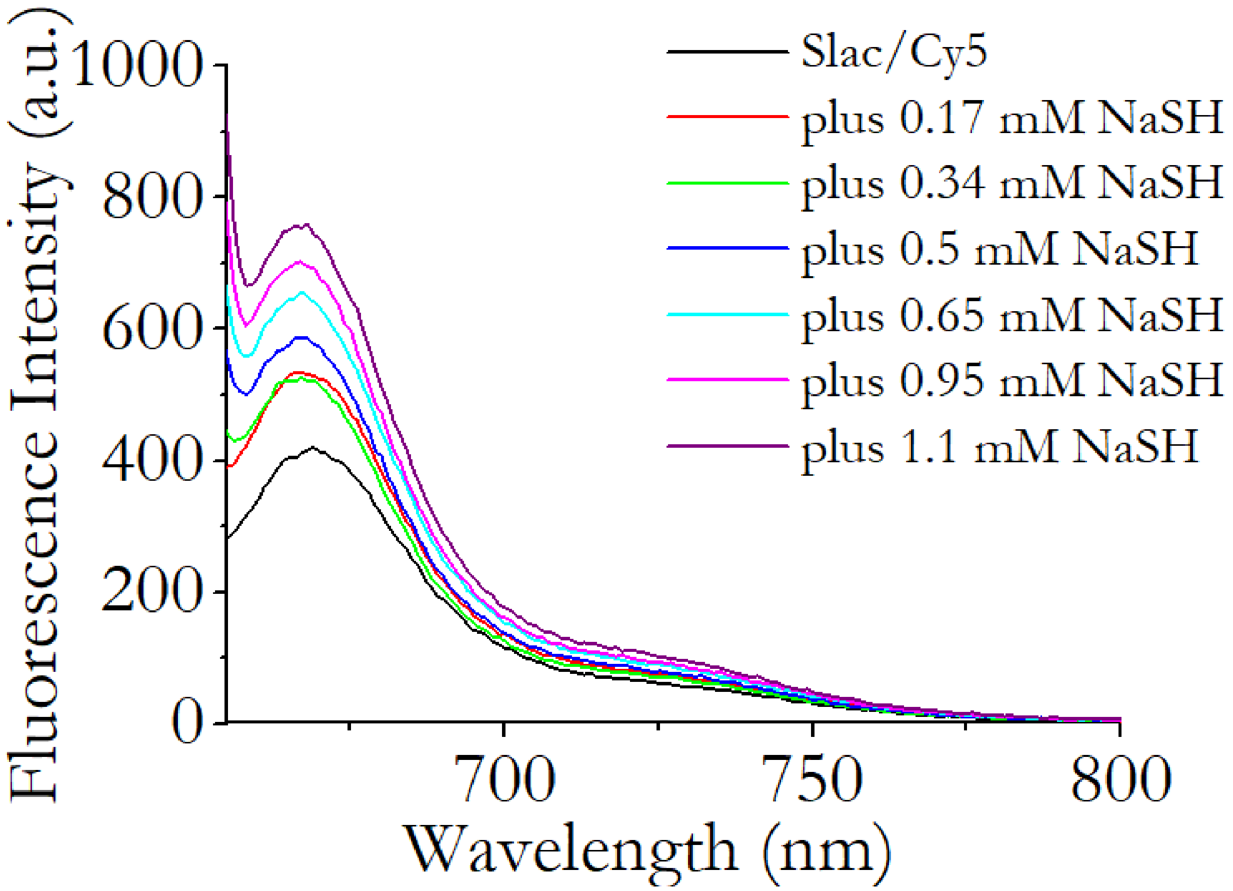

Figure S7. Emission spectra of SLAC_Cy5 before and after the addition of increasing amounts of NaSH. 100 mM potassium phosphate buffer (pH = 6.8); λ_{ex} = 651. Protein concentration: 120 nM.

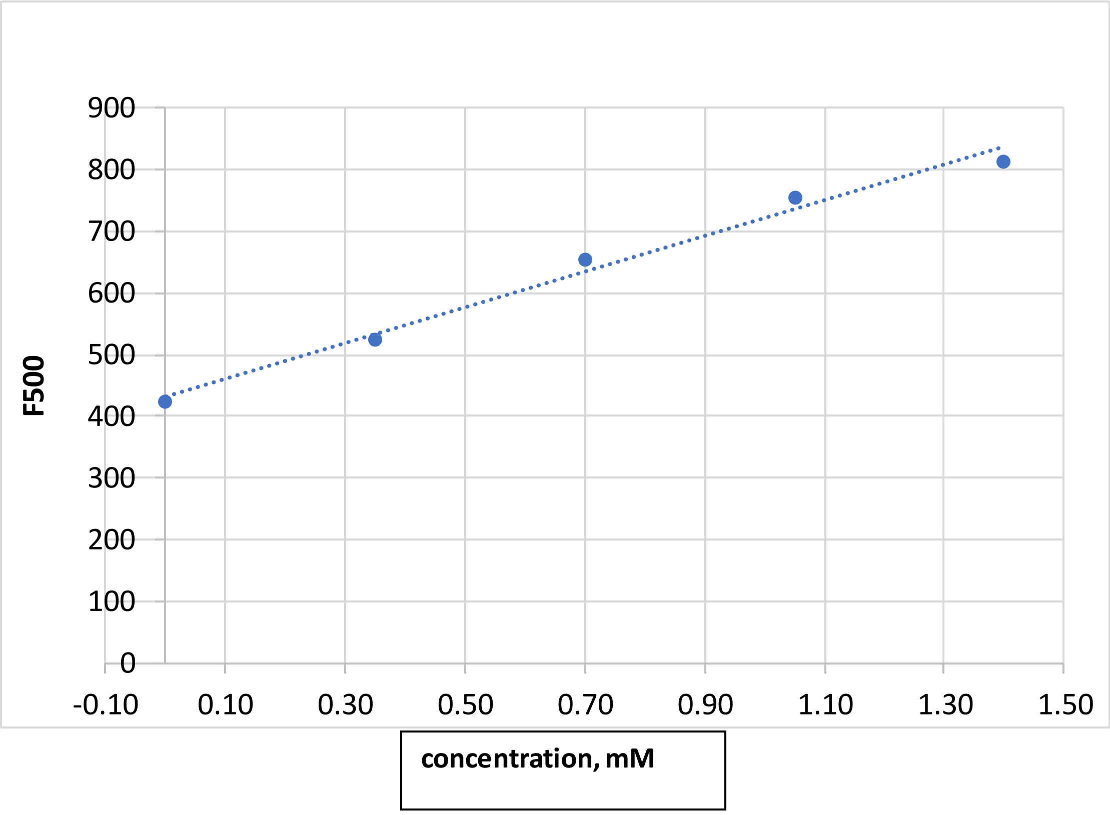

Figure S8. Calibration line of SLAC_Cy5 (; λ_{exc} = 570 nm; λ_{em} = 645 nm) for the addition of increasing amounts of HS^-.

Table S1. The standard deviation of the blank (σ), the absolute value of the slope of the calibration line (K) (Figure S9) and LOD value.

sample	σ	K	LOD (µM)
SLAC_Cy5	8	288 µM	90 µM

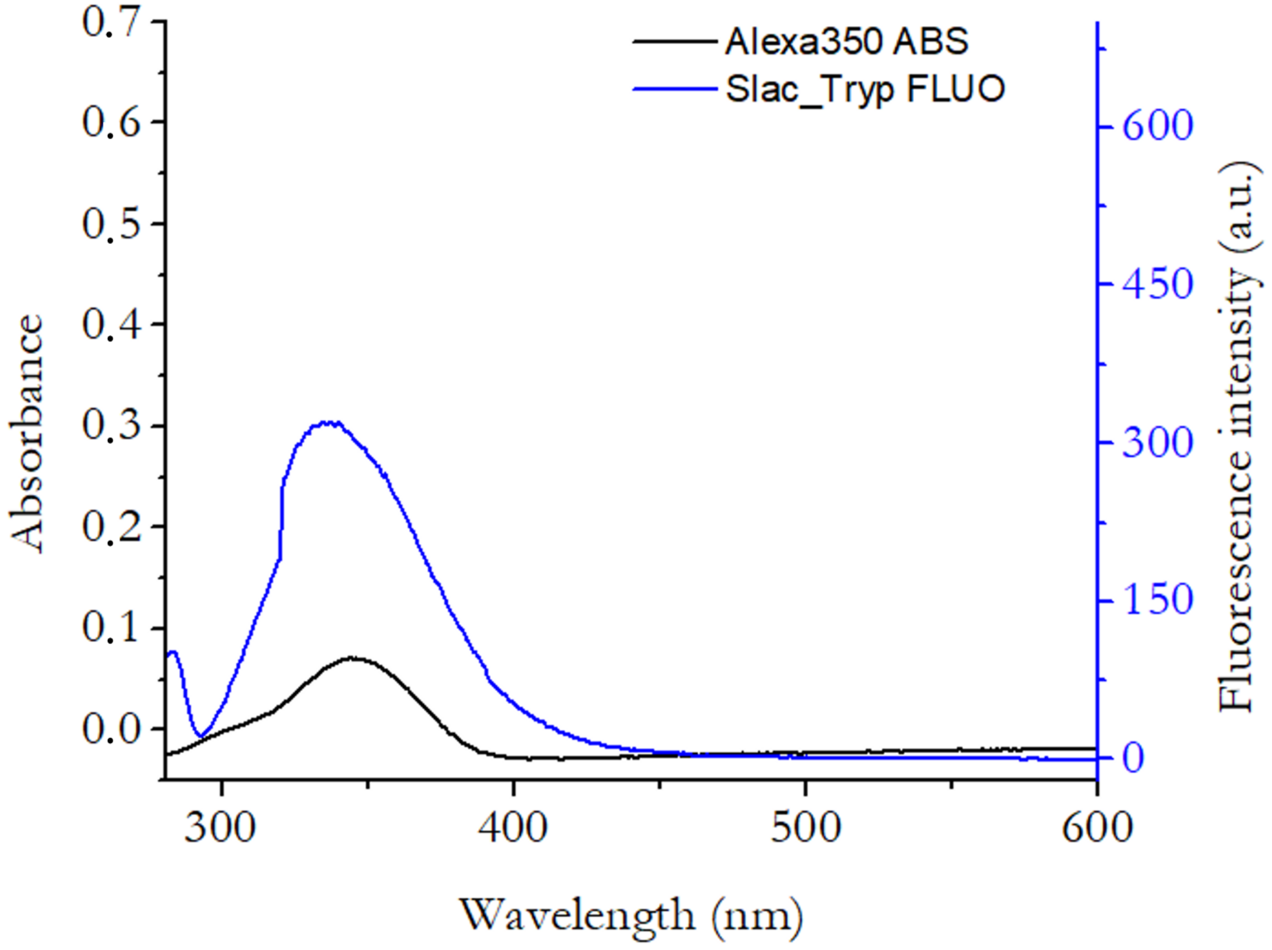

Figure S9. Overlay of the electronic absorption spectrum of Alexa350 with the fluorescence emission spectrum of Trps in SLAC. λ_{ex} = 280 nm. Protein concentration: 30 µM.

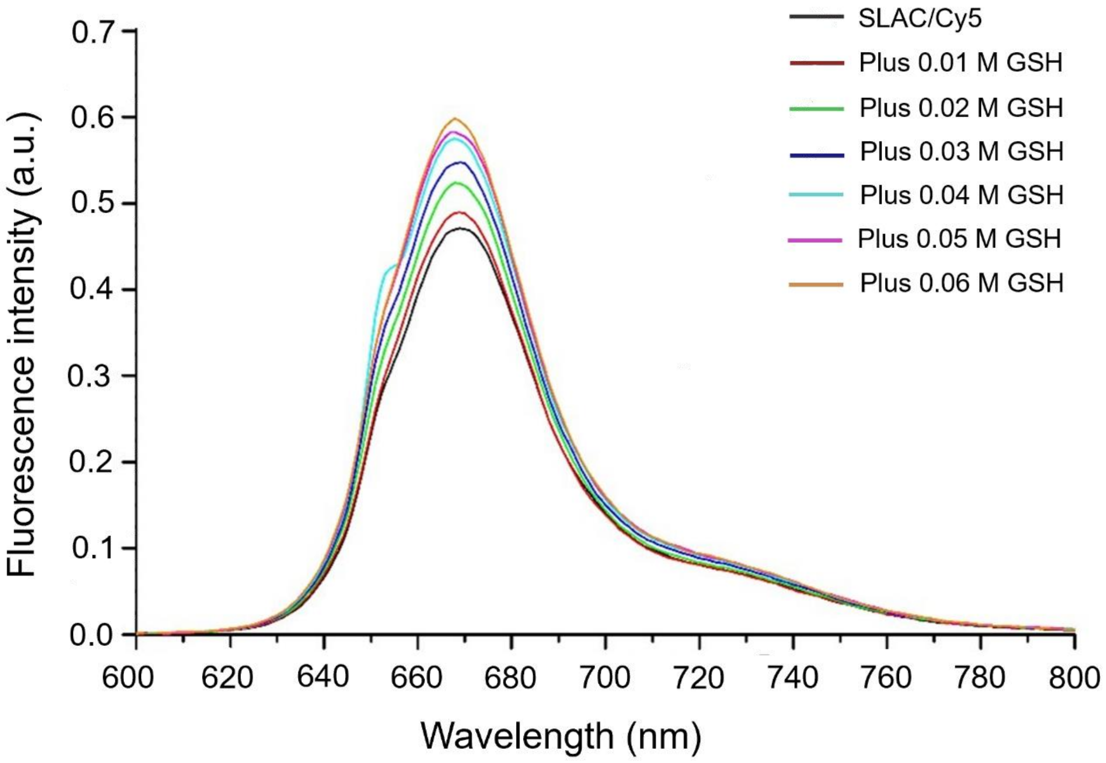

Figure S10. Room temperature fluorescence intensity traces of Cy5 labelled SLAC (exc 651 nm) titrated with GSH. Protein concentration: 120 nM in 100 mM potassium phosphate buffer (pH = 6.8).

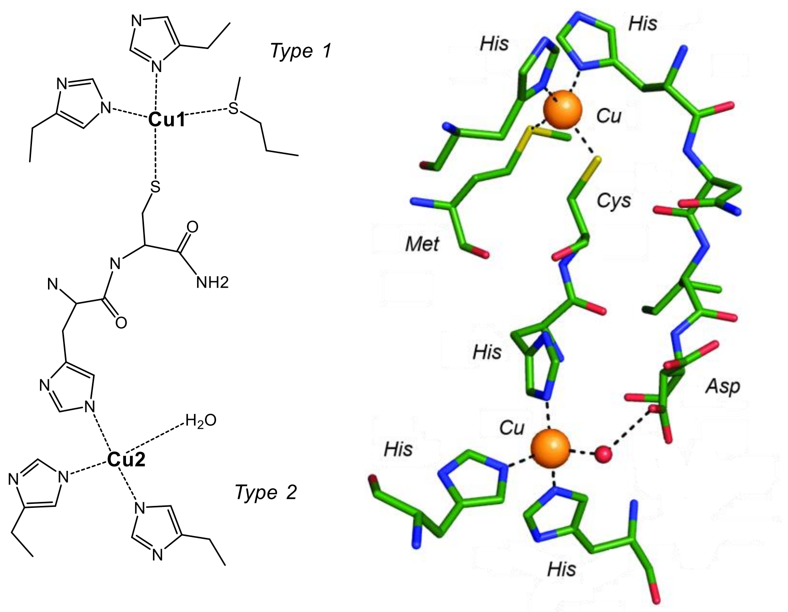

Figure S11. Schematic representation of the copper sites in NiR.

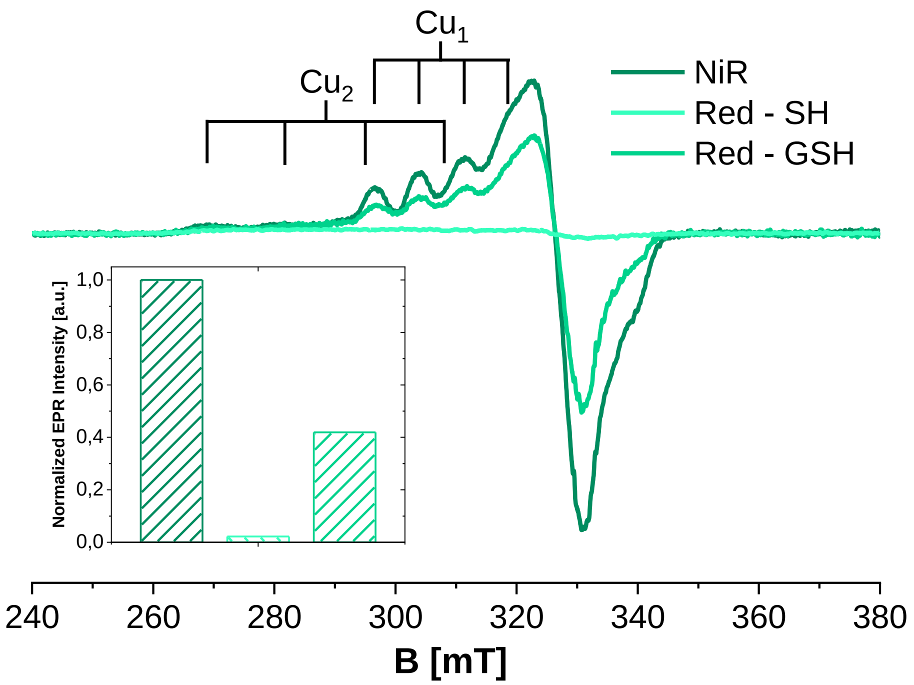

Figure S12. CW-X-band EPR spectra recorded at 77 K of the oxidized NiR and reduced with SH and GSH. The signal intensities reported in the inset were obtained by double integration of the CW-EPR spectra. Protein concentration: 0.13 mM with 30% glycerol.

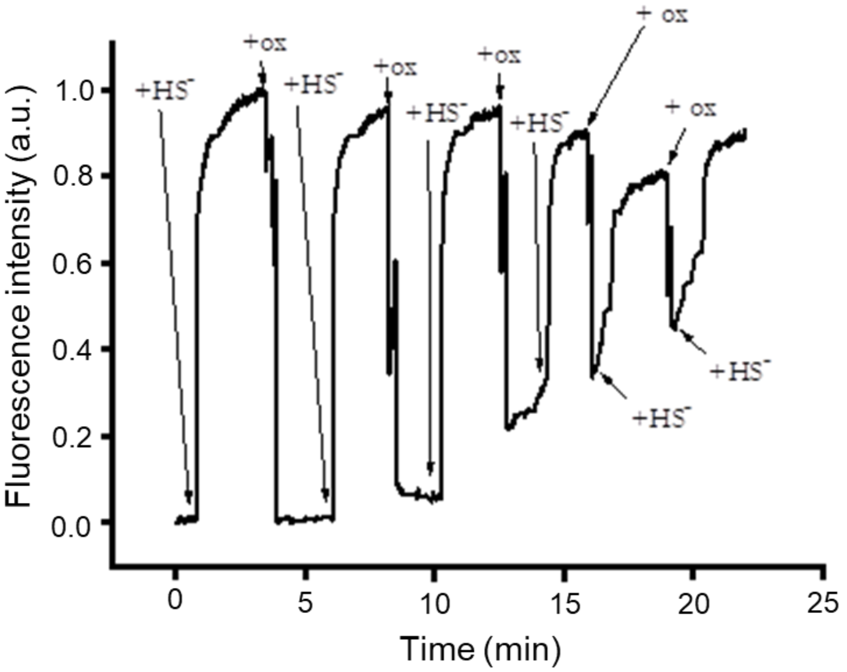

Figure S13. Fluorescence time trace for a solution containing Cy5-labelled NiR upon subsequent addition of NaSH (50 μM) and of excess of oxidant ($K_3Fe(CN)_6$). Protein concentration: 120 nM in 100 mM potassium phosphate buffer (pH = 6.8); λ_{ex} = 651, λ_{em} = 670 nm. The first arrow indicates the time of injection of NaSH into the cuvette sample solution, the second arrow marks the time point at which $K_3Fe(CN)_6$ was introduced into the solution.

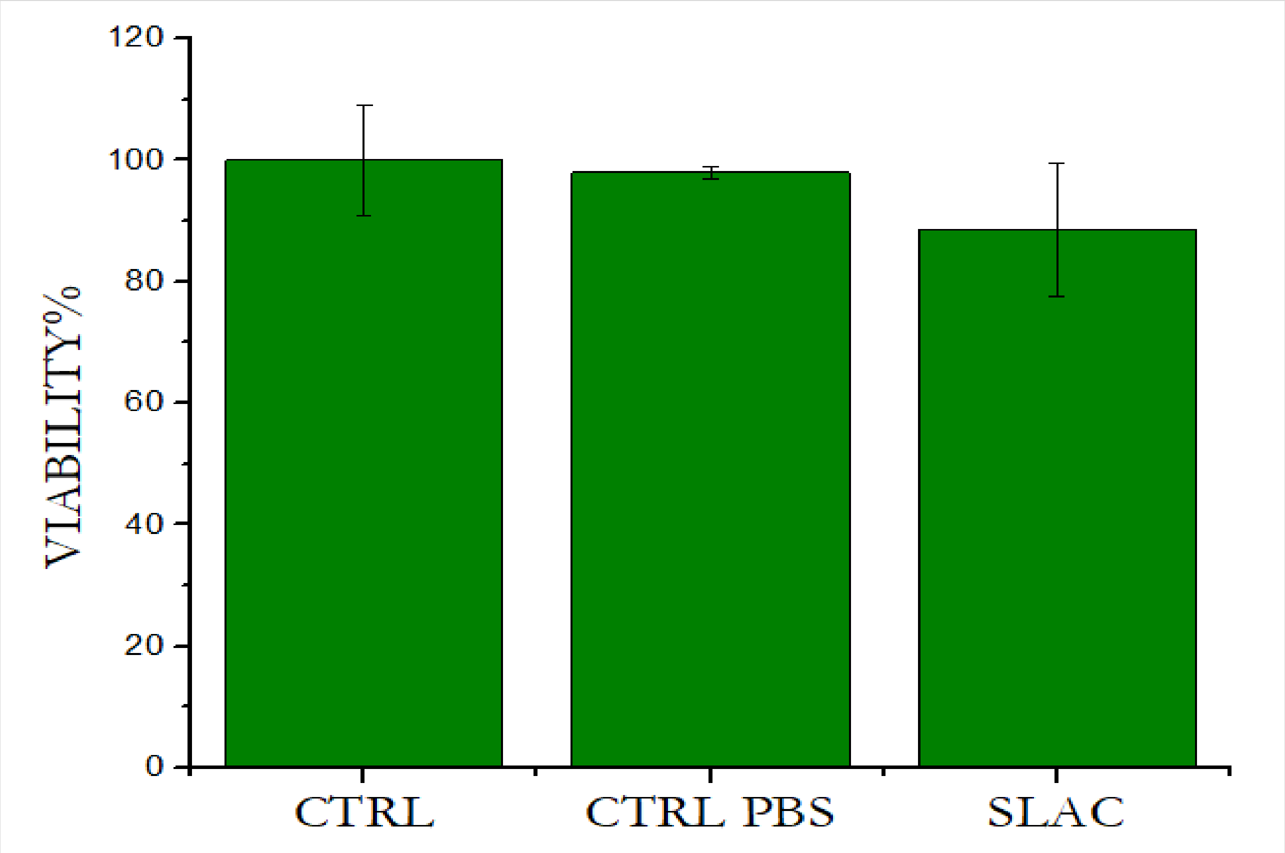

Figure S14. Cytotoxicity evaluation of SLAC_Cy5 in HepG2 cell line by MTT assay. Cells were incubated 2 h with SLAC_Cy5. Non-treated cells (Ctrl) and cells incubated with PBS (Ctrl PBS) were used as controls. Cell viability was compared with non-treated cells (Ctrl) and data were reported as the mean percentage ± standard deviation.

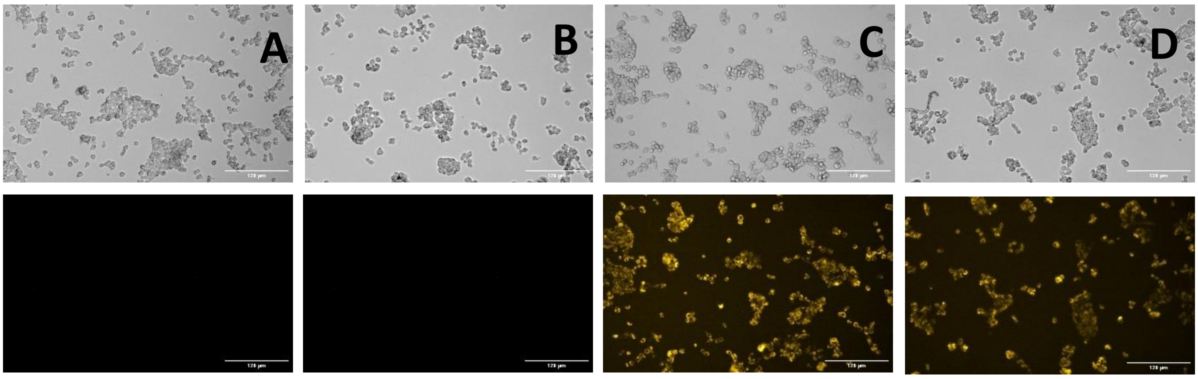

Figure S15. Fluorescence microscopy images of non-treated HepG2 cells (A), of HepG2 cells treated with PBS (B), of HepG2 cells incubated with BSA_Cy5 (C), of HepG2 cells incubated with BSA_Cy5 + 260 µM NaSH (exogenous HS^{-}) (D). Protein concentration: 120 nM. Magnification 20x.
